# Longitudinal analysis of the lung microbiota of cynomolgous macaques during long-term SHIV infection

**DOI:** 10.1186/s40168-016-0183-0

**Published:** 2016-07-08

**Authors:** Alison Morris, Joseph N. Paulson, Hisham Talukder, Laura Tipton, Heather Kling, Lijia Cui, Adam Fitch, Mihai Pop, Karen A. Norris, Elodie Ghedin

**Affiliations:** Division of Pulmonary, Allergy and Critical Care Medicine, Department of Medicine, University of Pittsburgh School of Medicine, Pittsburgh, PA USA; Department of Immunology, University of Pittsburgh School of Medicine, Pittsburgh, PA USA; Center for Bioinformatics and Computational Biology, University of Maryland, College Park, MD USA; Department of Biology, Center for Genomics & Systems Biology and College of Global Public Health, New York University, 12 Waverly Place, New York, NY 10003 USA; Department of Computational and Systems Biology, University of Pittsburgh School of Medicine, Pittsburgh, PA USA; Tsinghua University School of Medicine, Beijing, China; Present address: Department of Biostatistics and Computational Biology, Dana-Farber Cancer Institute, Boston, MA, USA; Present address: Department of Biostatistics, Harvard School of Public Health, Boston, MA, USA

**Keywords:** SHIV, Microbiota, 16S rRNA, Time series

## Abstract

**Background:**

Longitudinal studies of the lung microbiome are challenging due to the invasive nature of sample collection. In addition, studies of the lung microbiome in human disease are usually performed after disease onset, limiting the ability to determine early events in the lung. We used a non-human primate model to assess lung microbiome alterations over time in response to an HIV-like immunosuppression and determined impact of the lung microbiome on development of obstructive lung disease. Cynomolgous macaques were infected with the SIV-HIV chimeric virus SHIV_89.6P_. Bronchoalveolar lavage fluid samples were collected pre-infection and every 4 weeks for 53 weeks post-infection. The microbiota was characterized at each time point by 16S ribosomal RNA (rRNA) sequencing.

**Results:**

We observed individual variation in the composition of the lung microbiota with a proportion of the macaques having *Tropheryma whipplei* as the dominant organism in their lungs. Bacterial communities varied over time both within and between animals, but there did not appear to be a systematic alteration due to SHIV infection. Development of obstructive lung disease in the SHIV-infected animals was characterized by a relative increase in abundance of oral anaerobes. Network analysis further identified a difference in community composition that accompanied the development of obstructive disease with negative correlations between members of the obstructed and non-obstructed groups. This emphasizes how species shifts can impact multiple other species, potentially resulting in disease.

**Conclusions:**

This study is the first to investigate the dynamics of the lung microbiota over time and in response to immunosuppression in a non-human primate model. The persistence of oral bacteria in the lung and their association with obstruction suggest a potential role in pathogenesis. The lung microbiome in the non-human primate is a valuable tool for examining the impact of the lung microbiome in human health and disease.

**Electronic supplementary material:**

The online version of this article (doi:10.1186/s40168-016-0183-0) contains supplementary material, which is available to authorized users.

## Background

The lung microbiome is challenging to study longitudinally in health and disease. Whether the microbiome of the lung is stable over time is currently unknown as studies in humans have largely been limited to cross-sectional analyses due to the invasive nature of bronchoscopy or lung tissue collection. In addition, study of the lung microbiome in individuals with lung disease occurs primarily after disease is already manifest, limiting the ability to investigate changes in the microbiome that occur early in the disease process.

Animal models allow for serial sampling of the lung both before and after development of disease. Non-human primates (NHPs) such as rhesus macaques are useful models in microbiome studies for several reasons: (a) compared to other animal models, such as mice, the microbiome composition of NHP is more similar to the one seen in humans [1]; (b) NHPs are, like humans, outbred; (c) longitudinal measurements before and after infection or disease development can more easily be obtained than for humans; and (d) confounding variables such as smoking or diet can be controlled. While the monkey gut, oral cavity, and vagina have been surveyed for microbiome studies [2–4], no work has previously been done on the lung microbiome of NHPs. Here, we used cynomolgous macaques as an NHP model to determine longitudinal changes of the lung microbiome, including alterations occurring during a chronic HIV-like infection and in association with the development of chronic obstructive pulmonary disease (COPD).

Both local and systemic diseases may have an impact on the lung microbiome. For example, immune dysfunction that occurs in HIV infection could lead to microbial differences in the lung. A recent study demonstrated that *Tropheryma whipplei* was present more frequently in HIV-infected individuals than in HIV-uninfected, suggesting a correlation between bacterial lung residents and HIV infection [5]. Lung diseases such as COPD have been postulated to result in shifts in the lung microbiome [6–8], but the point at which these changes occur during the disease process is unknown. COPD is also a common complication in HIV-infected individuals, with estimates of a prevalence of 10–20 % depending on the population studies [9–11]. Macaques infected with a SIV-HIV chimeric virus (that replicates an HIV-like infection) and colonized with the opportunistic fungus, *Pneumocystis* (Pc), develop chronic airflow obstruction with radiographic and morphometric evidence of emphysema and lung airspace enlargement [12]. Using this model, we characterized bacterial microbiota changes over the course of SHIV infection and we correlated the bacterial microbiota with the development of COPD.

## Results

### Taxonomic composition of the lung microbiota

Using target gene sequencing, we analyzed the composition of the lung microbiota in the bronchoalvealoar lavages (BAL) of 12 cynomolgus monkeys at baseline and over 53 weeks post-infection with the SIV-HIV chimeric virus SHIV_89.6P_. The V1–V3 region of the 16S ribosomal DNA (rDNA) was PCR-amplified and sequenced on the Roche/454 GS-FLX sequencing platform. Reads were clustered into operational taxonomic units (OTUs) using DNAclust [13]. We identified 6002 OTUs present in more than one sample with 25 to 777 OTUs per sample (median = 341, average = 355); 82 % of the OTUs had significant matches to 736 distinct taxa from 189 genera. The top 9 genera present in the highest proportion in the BALs across monkeys, and across time points, include members of the phyla Proteobacteria (*Acinetobacter*, *Neisseria*, and *Uruburuella*), Bacteroidetes (*Porphyromonas*, *Prevotella*, and *Flavobacterium*), Fusobacteria (*Fusobacterium*), Firmicutes (*Streptococcus*), and Actinobacteria (*Tropheryma*).

**Fig. 1:**
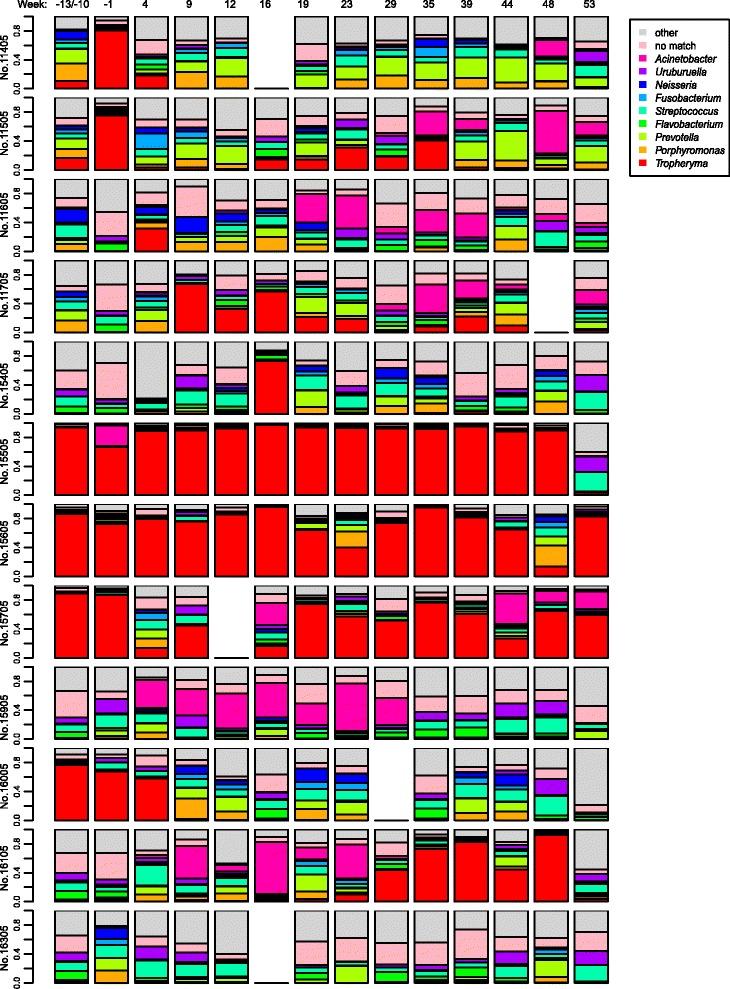
Proportion of main genera in bronchoscopic alveolar lavage (BAL). Each row is a monkey, and each column is a BAL sample. Time points pre- and post-SHIV infection are shown *above* each BAL. While the microbiota of different monkeys is highly divergent, within the same monkey the microbiota composition is relatively stable over time. *Tropheryma whipplei* (*red*) was the predominant organism in several monkeys

**Fig. 2:**
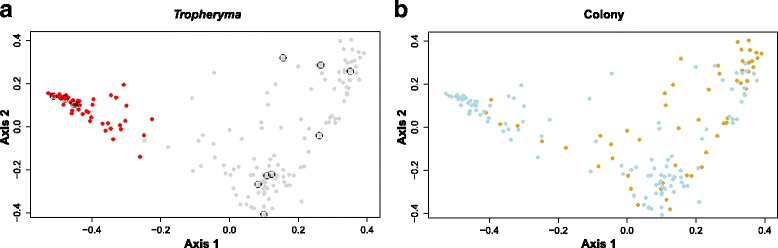
Principal Coordinate Analysis (PCoA). Each BAL sample is represented by a *dot* and the distances between dots represent the weighted UniFrac distance between samples. During PCoA, samples were scaled down to two unit-free dimensions (coordinates) for visualization. **a** *Tropheryma*-dominant vs high diversity monkeys. *Red dots* correspond to time points at which *Tropheryma whipplei* was the predominant organism in the BAL sample of any monkey; *circles* highlight the earliest time point for each monkey (weeks −13 or −10). **b** Monkeys color-coded by colony from which the monkey originated. *Yellow* and *blue dots* separate the samples collected at all time points from monkeys originating from each of the two colonies. Colony origin does not predict microbial composition, as seen by the lack of separation of the samples based on colony

Surprisingly, at baseline (time points –10 or –13 weeks before SHIV infection), a number of monkeys had BAL microbiota dominated by the genus *T. whipplei*, while other monkeys had high diversity in their microbiota with no one genus making up more than 25 % of the microbiota (Fig. 1). Within the higher diversity samples, the most prevalent genera (15–25 % relative abundance) were *Streptococcus*, *Prophyromonas*, *Neisseria*, and *Uruburuella. Tropheryma*-dominant monkeys had reduced alpha diversity (average Simpson’s Index 0.76, SD = 0.08) compared to high diversity monkeys (Simpson’s Index 0.97, SD = 0.03; Wilcoxon *p* = 0.009). These *Tropheryma*-dominant monkeys also exhibited altered microbiota composition at baseline as measured by adonis/PERMANOVA testing (*p* = 0.005) (Fig. 2a). Over the course of the infection, the majority of their samples continued to cluster away from high diversity monkeys (Fig. 2a). For both the *Tropheryma*-dominant and high diversity monkeys, there was higher microbiota variation observed between monkeys (adonis *p* is <0.001) at each time point than between time points for the same monkey (variation over time *p* = 0.145).

Because the monkeys were obtained from two different colonies, we tested for a batch effect at baseline. Colony origin did not affect alpha diversity (Wilcoxon *p* = 0.153), but did affect composition (adonis/PERMANOVA *p* = 0.004); the *Tropheryma*-dominant monkeys, for example, were found to be from the same colony. However, these monkeys did not cluster over the course of the study (Fig. 2b).

### Taxa associated with SHIV

To explore the effect of SHIV infection on the lung microbiota, we compared pre-infection to all post-infection samples. We identified no taxonomic group that had strong statistical association to SHIV status (metagenomeSeq *p* < 0.05 and log2 fold change >2). When relaxing our log2 fold change threshold, we observed one OTU differentially abundant. In particular, OTU 4802 annotated as *Uruburuella suis* was found to be more abundant in pre-infected samples with a log2 fold change of −1.79. Furthermore, we did not observe any significant correlation between microbiota profiles and CD4 or SHIV viral levels (data not shown).

### Association of lung microbiota with chronic obstructive pulmonary disease (COPD)

**Fig. 3:**
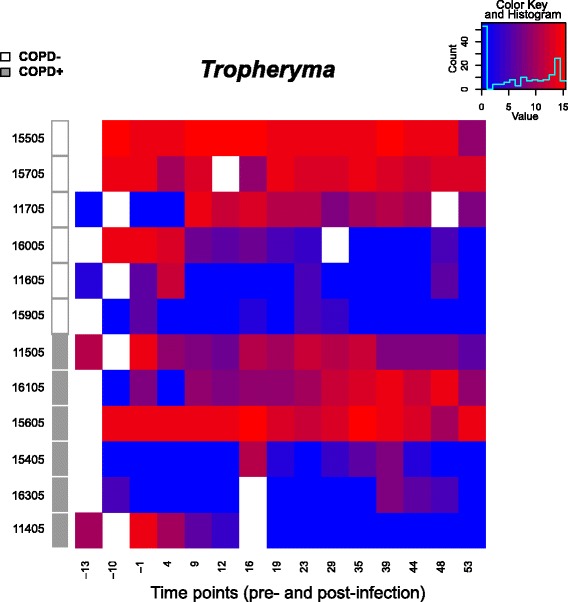
*Tropheryma whipplei* abundance across time and clinical outcome. Abundance ranges from low (*blue*) to high (*red*) with *white* indicating missing data. Monkeys were grouped by COPD status. Within each group, they are clustered by the abundance profile of *Tropheryma. White* and *grey boxes* on the *left side* indicate COPD status. *Tropheryma* abundance was not associated with COPD

**Fig. 4:**
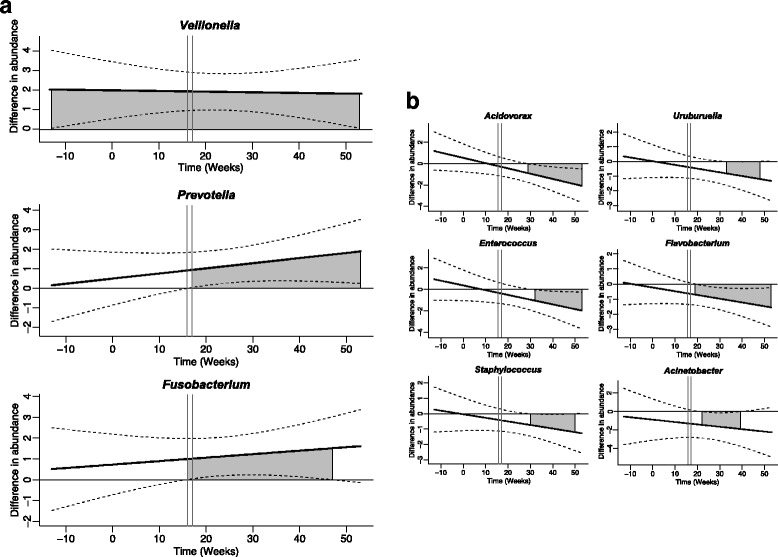
Smoothing Spline ANOVA (SSANOVA) showing progression of differential abundance of specific oral microbial taxa during the course of infection. The *y*-axis represents the difference in normalized abundances of these taxa between monkeys that developed COPD and those that did not. All monkeys were used in this analysis. Values above zero indicate an enrichment of the specific taxon in monkeys that develop COPD. *Dashed lines* are the confidence intervals. The *shaded area* indicates time when the differences in abundance of each taxon are significant as compared to baseline abundance (of the control group). The *x*-axis represents time in weeks pre-SHIV and post-SHIV infection. We highlight with *vertical lines* weeks 16 and 17 when there was a decrease in pulmonary function in monkeys that developed COPD. **a** Taxa positively associated with COPD. **b** Taxa negatively associated with the disease

We compared the lung microbiota of monkeys that developed obstructive pulmonary disease (determined by increased airway obstruction on pulmonary function testing) during the study period with that of monkeys that did not (Table 1). We first investigated the potential relationship of *T. whipplei* frequency and prevalence to COPD, but did not observe an enrichment of this organism within the COPD group (Fig. 3). To determine associations of other OTUs with COPD, we then used linear mixed models and time-dependent Smoothing Spline ANOVA (SSANOVA) (Additional file 1: Tables S1 and S2). We found that OTUs belonging to several oral microbial taxa—*Fusobacterium*, *Prevotella*, *Veillonella*, *Neisseria*, and *Porphyromonas*—were enriched in the BALs of animals that developed COPD (Fig. 4). Several potential respiratory pathogens such as *Uruburuella suis*, *Streptococcus sp.*, and *Flavobacterium* sp. were enriched in the animals that did not develop COPD.

**Table 1:** Demographic characteristics of macaques by chronic obstructive pulmonary disease (COPD) status

	COPD+ (*n* = 6)	COPD− (*n* = 6)	*p* value
Age, mean (SD)	7.3 (2.9)	8.8 (4.8)	0.53
Female, *n* (%)	2 (33)	2 (33)	1.00
Peak viral load, mean RNA copies per ml (SD)	4.7 × 10^7^ (8.8 × 10^7^)	3.3 × 10^7^ (2.3 × 10^6^)	0.70
Acute phase (4 wpi) CD4+ T cells, mean (SD)	816 (663)	370 (276)	0.16
Chronic phase (20 wpi) CD4+ T cells, mean (SD)	888 (622)	705 (434)	0.57

*wpi* weeks post-infection

To further explore this association, we employed SSANOVA to investigate the time-related changes in the lung microbiota in the context of airway obstruction. This test allows us to determine not only whether a difference exists but also at which time point the difference becomes manifest. *Veillonella* was more abundant over the entire study period in the lungs of monkeys that eventually developed COPD, while *Prevotella* and *Fusobacterium* exhibited an enrichment only starting at weeks 16 and 17 post-SHIV infection (Fig. 4a). This period is consistent with the time when persistent declines in pulmonary function were evident in the monkeys. Several other taxa appeared by SSANOVA to be associated with COPD, including *Treponema*, *Catonella*, and *Granulicatella* (Additional file 1: Table S2). Taxonomic groups enriched in non-COPD samples included *Flavobacterium* and *Uruburuella*, consistent with the cross-sectional statistical findings. In addition, we noted a decrease in *Acinetobacter*, *Staphylococcus*, *Enterococcus*, *Acidovorax* (Fig. 4b), and *Kineosporia* (Additional file 1: Table S2). Although detection of the fungus *Pneumocystis* in the lungs has been shown to be associated with COPD in this animal model, we did not find any changes in bacterial taxa according to *Pneumocystis* colonization status (data not shown).

#### Network view of SHIV+ lung microbiota

**Fig. 5:**
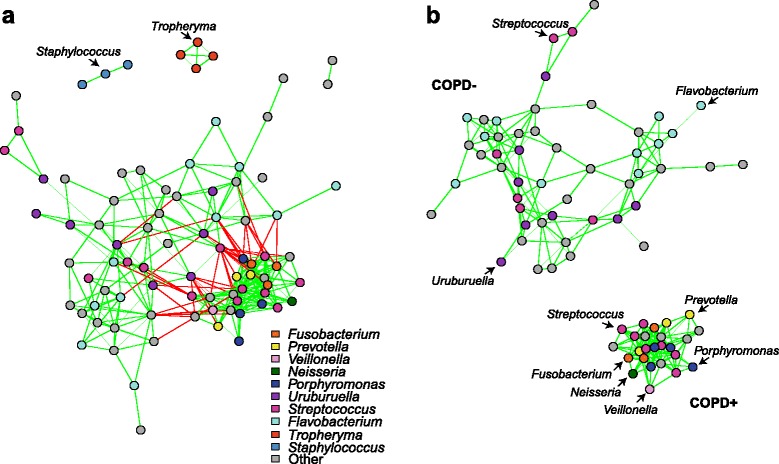
Correlation network analysis using SPIEC-EASI. **a** The ecological correlation network shows two groups of OTUs that are negatively correlated with each other. One group includes OTUs identified as enriched in COPD animals (COPD+) and the other includes OTUs enriched in non-COPD animals (COPD−). Each node represents an OTU and is colored by its assigned taxonomy. *Green edges* represent positive correlation between OTUs and *red* represent negative correlation. **b** Negative edges have been removed to show that the two groups have no positive correlations with each other. This separation emphasizes the shift in community composition that occurred when an animal developed COPD

To determine whether there was an organizational principle for the microbial community within the lung of the monkeys over the course of SHIV infection, we investigated inter-microbial species associations by correlation network analysis using the differential abundance data. We built this network with SParse InversE Covariance estimation for Ecological ASsociation Inference (SPIEC-EASI) [14]. The network demonstrated negative associations between two large groups of OTUs and positive associations within each group (Fig. 5a); singleton OTUs (i.e., not associated with any other OTU) were removed. In the largest group, we identify OTUs belonging to the genera that were seen by SSANOVA to be enriched in animals that developed COPD including *Fusobacterium*, *Prevotella*, *Veillonella*, *Neisseria*, and *Porphyromonas* (Fig. 5b). In the smallest group, we saw OTUs in the genera identified as enriched in non-COPD animals, including *Uruburuella* and *Flavobacterium* (Fig. 5b). Other OTUs in this group that were not identified by the other methods belonged to genera including *Kinesporia*, *Enterococcus*, and *Vibrio*. Most interestingly, OTUs belonging to the *Streptococcus* genus were found in both groups, highlighting its importance within both COPD and non-COPD bacterial communities. The separation of the two groups showed the difference in community composition that accompanied the development of COPD. The negative correlations between members of the two groups emphasize how shifts in one species can impact multiple other species, potentially resulting in disease.

## Discussion

We performed a longitudinal sampling of the lung microbiota in non-human primates over 53 weeks post-SHIV infection. We found that there was a high degree of variation in the microbiota of individual animals with two apparent community types—one dominated by *T. whipplei* and the other characterized by greater microbial diversity. Although there were shifts in the bacterial communities of individual monkeys over time, the community structure of an individual animal was relatively consistent. We found that there were minimal changes over the course of an HIV-like immunosuppression and that relative increases in oral bacteria predominated during development of COPD.

Non-human primates are a highly relevant model for the study of the lung microbiome. Because monkeys are outbred and have an upright posture, their lung microbiome is likely more similar to humans than to mice. In addition, the model overcomes difficulties encountered in human lung microbiome studies such as the inability to perform frequent lower lung sampling, the influence of other factors such as smoking, diet, and medication use, and easier access to the lung with potentially less oral contamination. This study is the first to report on longitudinal changes in the lung microbiota in any animal model. Although human studies have reported serial bronchoscopies or frequent sputum specimens, performance of monthly bronchoscopies is not possible in humans; thus, this study provides data that have direct relevance to the study of the human lung microbiome. In the lung, there were greater differences between animals than within the same animal over time; however, individual animals did have samples that differed from their baseline community structure at certain time points. Other studies have shown that the stability of the microbiome is dependent on the body site sampled, with the oral microbiome being less stable over time than, for example, the vaginal microbiome [15]. Similar longitudinal analyses have not been done for the lung.

The monkeys were infected with SHIV and developed a HIV-like immunosuppression over the course of the study. We were thus able to study pre- and post-SHIV infection, which is challenging in human HIV studies. This allowed us to evaluate shifts in the bacterial composition of the lung prior to and after infection, as well as during development of COPD, defined as development of fixed airway obstruction. While we found alterations in several bacteria, no large shifts were observed in specific bacterial taxa during the course of SHIV infection. Similar findings have been reported by the Lung HIV Microbiome Project that described changes in the oral microbiome in HIV-infected individuals compared to HIV-uninfected controls, but fewer differences in the lung microbiota [16]. Whether changes in the lung microbiome occur prior to, or during, opportunistic lung infections or with profound immunosuppression in this animal model of human disease is unknown.

Interestingly, some monkeys had in their lungs a predominance of *T. whipplei*, the etiologic agent of Whipple’s disease, which persisted over the course of the study while others demonstrated transient colonization. These findings may be similar in humans where some individuals have been reported to have high levels of *T. whipplei* in BAL, although whether *T. whipplei* persists is not known [5, 17, 18]. In a recent study, *T. whipplei* was reported to be more prevalent in individuals who smoked or were HIV-infected [5], while another study showed no correlation with HIV [19]. In our non-human primate model, we found that *T. whipplei* was actually more common prior to SHIV infection. These differences may result from environmental variations, may be due to biological differences between monkeys and humans or a result from random sampling. There was also no relationship of *T. whipplei* to the development of COPD in the animals.

We found by cross-sectional statistics and by network analysis that several oral microbial taxa, such as *Fusobacterium*, *Prevotella*, *Veillonella*, *Neisseria*, and *Porphyromonas*, were enriched in the BALs of SHIV-infected animals that developed COPD. Oral bacteria have previously been implicated in COPD pathogenesis in humans who smoke [18], but have not been reported in an HIV-associated model. Furthermore, changes in the lung prior to development of COPD in humans have not been assessed. With the exception of *Neisseria*, all these organisms are anaerobes and have been associated with periodontal diseases and infections of the respiratory tract [20–22]. While specific oral bacteria have previously been found to be associated with airway obstruction in smokers [18], they had not yet been reported in a non-smoking population. These alterations in bacterial prevalence suggest that failure to clear oral anaerobes after aspiration may be a common mechanism in these types of lung diseases. Whether treatment of these organisms would prevent or improve COPD is unknown.

There are several limitations of the study. First, as with any study of the lung microbiome, we are examining a low biomass sample and contamination is always of concern. We also could not account for bronchoscopic carryover from the mouth as we did not collect oral samples in parallel. As the monkeys are deeply sedated at the time of bronchoscopy, insertion of the bronchoscope is easier, limiting contact with the oral cavity. Second, we are primarily focusing on the bacterial taxonomy of the lung. Viruses or fungi may play a role, and functional aspects of the microbiome are likely important.

## Conclusions

In summary, this study is the first to contribute detailed information on monthly changes in the lung over a long time period in a model directly relevant to humans. The lung microbiome demonstrated variability between normal animals at baseline with relatively less variation over time, despite development of immunosuppression. The study is also the first to investigate changes in the lung prior to development of COPD in a non-smoking model and found that persistence of oral microbes were associated with the development of COPD. The exact role of these bacteria in COPD pathogenesis is currently unknown, but similar data in humans suggest a potential mechanistic role. The lung microbiome in the non-human primate is a valuable tool for examining the impact of the lung microbiome in human health and disease.

## Methods

### Animals

Adult Chinese-origin, cynomolgus macaques (*Macacca fasicularis*) were procured from the University of Pittsburgh Department of Laboratory Animal Research-approved vendors. Macaques were housed in the University of Pittsburgh non-human primate facility, which is accredited by the American Association for Accreditation of Laboratory Animal Care. Animal care and husbandry was provided in accordance with standards set forth in the *Guide for the Care and Use of Laboratory Animals* [23]. All experimental animal procedures were approved by the University of Pittsburgh Institutional Animal Use and Care committee (approval number 08103308) prior to the start of the study, and were in full compliance with the Provisions of the Animal Welfare Act, and other applicable laws and regulations.

### SHIV infection

Cynomolgus macaques (*n* = 12) were inoculated with SHIV_89.6P._ All were continuously exposed to *Pneumocystis* via cohousing with simian immunodeficiency virus (SIV) and *Pneumocystis* co-infected macaques. Natural airborne transmission of *Pneumocystis* in this model system has been previously described [12, 24]. Demographic characteristics of animals are given in Table 1. Pulmonary function tests were performed at baseline (pre-SHIV infection) and every other month after SHIV infection (up to 12 months post-infection) by use of whole body plethysmography and forced deflation technique on anesthetized monkeys, as described [12]. SHIV-infected monkeys that became colonized with *Pneumocystis* (*n* = 6) had evidence of obstructive pulmonary disease, which was defined by a decrease in the post-bronchodilator forced expiratory volume in 0.4 s [12]. Forced expiratory volume was obtained by performing whole body plethysmography, similar to human pulmonary function; decreases in this measure indicate development of airway obstruction. These animals also developed emphysema as determined by quantitative high resolution computed tomography scanning, and tissue morphometry [12].

### Lung microbiome sampling

Prior to each bronchoscopy, the bronchoscope was washed using Enzol enzymatic detergent (Johnson and Johnson Medical, New Brunswick, NJ), rinsed with water, and placed in a sterilizing bath of 2.5 % glutaraldehyde solution for at least 15 min. The bronchoscope was passed through the laryngeal folds and directed into the right main stem bronchus without suction. Once wedged, four aliquots of 10 ml sterile saline were lavaged. Bronchoalveolar lavage (BAL) fluid was immediately placed on ice and transported to the laboratory for processing. Unfractionated BAL fluid was filtered through a 40-μm-pore-size cell strainer (BD Falcon, Franklin Lakes, NJ). Lung cells were pelleted (5 min at 500*g*), and supernatant fluid was collected and stored at −80 °C until use.

### Sample processing and sequence library preparation

Raw BALs supernatants were spun at 10,000*g* and frozen at −80 °C. Pellets were resuspended in 180 μl of freshly prepared lysozyme solution (20 mg/ml lysozyme, 20 mM Tris-Cl, pH 8.0, 2 mM Na-EDTA, 1.2 % Triton X-100, filtered through a 0.22-μm filter) and incubated at 37 °C for 10 min. Extraction followed the Qiagen DNEasy Blood and Tissue kit protocol. Reagent controls, without BAL, were also processed through the Qiagen procedure. Extracted DNA was used in a PCR reaction with Roche/454 GS-FLX sequencing adapter fusion primers targeting the V1–V3 region of the bacterial 16S ribosomal RNA (rRNA). The reaction mix consisted of 0.75 U Accuprime Taq Hifi, 1× Accuprime PCR buffer II, 1 μl DNA sample, and 200 nmol of barcoded primer. Cycling conditions were 95 °C for 2 min, then 30 cycles of 95 °C for 20 s, 56 °C for 30 s, and 72 °C for 5 min. Reactions were cleaned up using 36 μl of Ampure Beads (Agencourt) and eluted into 25 μl low TE, pH 8.0. Eluted PCR products were quantitated in triplicate using DNA Picogreen (Invitrogen). Two final sample pools were created by combining 103 and 104 total PCRs; each positive sample had 20 ng added, while each negative control sample (buffer) had 20 μl added. The combined pools were then purified on a MinElute PCR column (Qiagen) and eluted into TE buffer. A total of 170 BAL samples were multiplexed in this manner in two separate gasket wells.

### Sequencing and data analysis

Sequencing was performed on the Roche/454 GS-FLX sequencer using Titanium chemistry (1,034,190 total raw sequences), with an average read length of 400 bp. Sequences that were too short (<75 cycles of the 454 instrument) or that contained at least one unrecognizable barcode (‘N’) were filtered from the data. Barcoded sequences were deconvoluted and reads were trimmed. Sequencing of PCR-amplified 16S rRNA genes resulted in 668,353 reads passing quality checks. Reads were clustered into 132,234 operational taxonomic units (OTUs) using DNAclust [13]. Only 6002 OTUs were detected in more than five samples or represented at least 20 sequences in a single sample and were included in further analysis. The number of OTUs per sample ranged from 25 to 777, with a median of 341 and an average of 355. The mean OTU size was 77, ranging from 5 to 59,920 with a median of 18 sequences. Representative sequences from the 6002 OTUs matched 736 distinct taxa from 189 genera. Among these, 1104 (18 %) did not have good matches (>100 bp exact match, >97 % identity) to isolate sequences from the RDP database (version 10.4). These were flagged as ‘no match’ in our analysis and assigned an OTU identifier. The resulting data were organized in a collection of tables at different taxonomic levels containing each taxonomic group as a row and each sample as a column. These tables formed the substrate for statistical analyses.

### Statistical analyses

Differential abundance was assessed with the Bioconductor package, metagenomeSeq [25]—a statistical method to incorporate the effect of under-sampling on the observed counts. Significant findings were reported for OTUs that satisfied the following criteria: (i) OTU had log2 fold change exceeding 2 in either cases or controls except in testing obstruction where we relaxed our threshold to a log2 fold change of 1; and (ii) statistical association was significant (*p* < 0.05) after Benjamini-Hochberg correction for multiple testing [26]. To test the shifts in abundance for specific taxa due to COPD, we employed Smoothing Spline ANOVA (SSANOVA) methods. To test for differentially abundant time intervals, we fit a smoothing spline on the difference in abundance between animals that developed COPD and animals that did not, and calculated 95 % Bayesian confidence intervals under the SSANOVA framework. This procedure estimates a function of the difference in abundance across time for each taxon. The SSANOVA procedure also included time, obstruction status and their interaction (http://cbcb.umd.edu/software/metagenomeSeq) [27]. We assumed an alpha of 0.2. Analyses were performed using the R software package 3.0.1 (http://www.R-project.org/) and metagenomeSeq 1.12.0 [25]. We fit a linear mixed model for each OTU independently to account for individual monkey variability while controlling for sampling day. We also included the cumulative sum scaling (CSS) normalization factor as a covariate in our linear mixed models. In the significance testing for our models, we retained only OTUs where the estimated Wald-type confidence intervals agreed in sign with each other. For fitting the models, we used the R package lme4 version 1.1-8 [28, 29].

### Ecological network construction

Ecological correlation networks were constructed on the 94 OTUs present in more than one-third of the samples (56 or more samples). Each network was built using SParse InversE Covariance estimation for Ecological ASsociation Inference (SPIEC-EASI) [14]. The final network was selected by random subsampling and interaction re-estimation using the Stability Approach to Regularization Selection (StARS) [30] with a 0.1 variability threshold. For visualization, OTUs outside the connected component were removed.

## Abbreviations

BAL, bronchoalveolar lavage; COPD, chronic obstructive pulmonary disease; HIV, human immunodeficiency virus; OTU, operational taxonomic units; PCoA, Principal Coordinate Analysis; rRNA, ribosomal RNA; SIV, simian immunodeficiency virus; SPIEC-EASI, SParse InversE Covariance estimation for Ecological ASsociation Inference; SSANOVA, Smoothing Spline ANOVA; StARS, Stability Approach to Regularization Selection

## Additional file

Additional file 1: Table S1. Estimates from a linear mixed model comparing taxa abundance between COPD+ and COPD− samples. Fold change estimates and basic statistics of several OTUs comparing COPD+ and COPD− samples 20 days after infection. In the linear mixed model, we accounted for individual monkey effects, days sampled, and included normalization factors. We filtered OTUs to those with at least 100 counts and declared OTUs significant if lower and upper log fold change estimates agreed in directionality. Row colors represent enrichment, green in COPD− and red in COPD+ samples. **Table S2** Smoothing Spline ANOVA estimates comparing COPD+ and COPD− taxa abundances through time. Smoothing Spline ANOVA estimates of the difference in genera abundances between COPD+ and COPD− groups through time. To test for differentially abundant time intervals, we fit a smoothing spline and calculated 95 % Bayesian confidence intervals under the SSANOVA framework. The SSANOVA procedure also included time, obstruction status and their interaction. We estimate significance using a permutation procedure and calculate significance with area under the curve as our test statistic. Row colors represent enrichment, green in COPD− and red in COPD+ samples. (XLSX 52 kb)
